# Sub-Picosecond Optical Dynamics in Perovskite NdNiO_3_ Thin Films via Broadband Pump–Probe Ellipsometry

**DOI:** 10.1021/acsphotonics.6c01634

**Published:** 2026-10-06

**Authors:** Yael Gutiérrez, Ranjan Kumar Patel, Saúl Vázquez-Miranda, Mateusz Rebarz, Shirly Espinoza, Yeonghoon Jin, Mikhail A. Kats, Shriram Ramanathan

**Affiliations:** † Departamento de Física Aplicada, 16761 Universidad de Cantabria, Avenida de los Castros, s/n, 39005 Santander, Spain; ‡ Department of Electrical and Computer Engineering, 242612 Rutgers University, Piscataway, New Jersey 08854, United States; § ELI Beamlines Facility, 528283 The Extreme Light Infrastructure ERIC, Za Radnicí 835, 25241 Dolní Břežany, Czech Republic; ∥ Department of Electrical and Computer Engineering, 5228 University of Wisconsin-Madison, Madison, Wisconsin 53706, United States

**Keywords:** pump–probe spectroscopy, spectroscopic ellipsometry, nickelate, correlated oxide, ultrafast, coherent phonons

## Abstract

The tunable physical properties of strongly correlated oxides make them interesting for emerging device applications in opto-electronics as well as serve as prototypical systems for condensed matter physics research. Here, we report room-temperature, broadband time-resolved spectroscopic ellipsometry (TRSE) measurements of ultrafast optical dynamics in perovskite nickelate NdNiO_3_ (NNO) thin films following femtosecond photoexcitation at three pump wavelengths (266, 400, and 800 nm). By tracking the transient complex dielectric function with sub-picosecond temporal resolution, we disentangle electronic and lattice contributions to the photoinduced response. Within the first picosecond, photoexcitation drives a rapid change in the dielectric function of NNO, attributed to the generation of a nonequilibrium electron population, accompanied by a transient reduction in Drude spectral weight and enhanced carrier scattering consistent with a crossover toward a bad-metal state. Short-lived coherent optical phonon oscillations are observed in the photon energy domain during the first hundreds of fs. At longer time scales (10–100 ps), coherent acoustic strain generates Brillouin oscillations in the transient ellipsometry signal, consistent with time-domain Brillouin scattering from the propagating strain pulse generated at the NNO layer after its transmission into the substrate.

## Graphical abstract

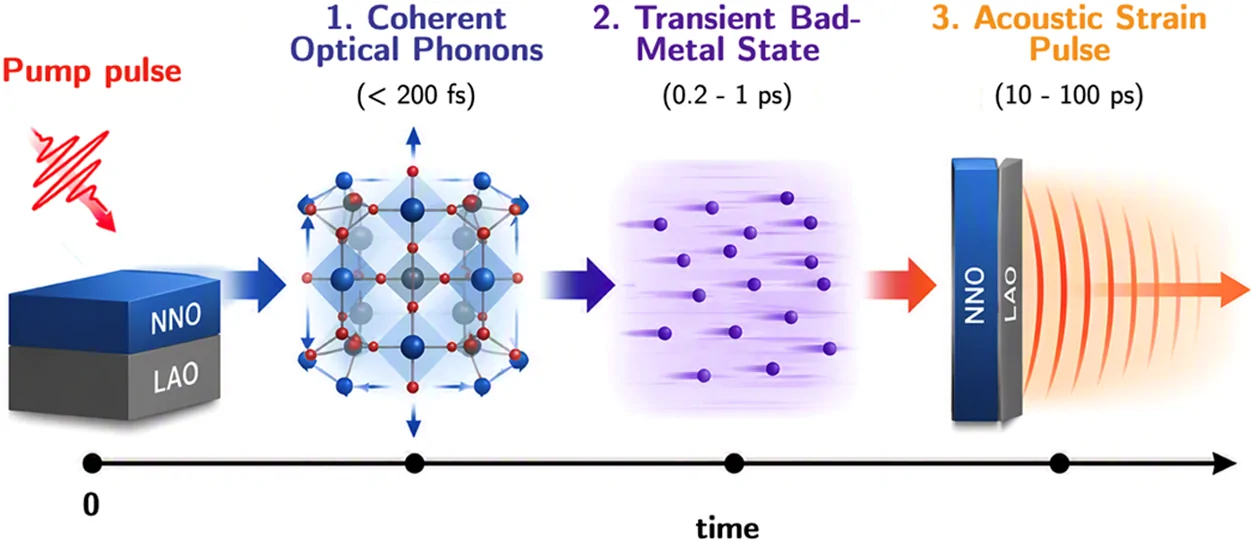

## Introduction

Ultrafast pump–probe spectroscopy provides femtosecond access to carrier dynamics, phonon excitations, and nonequilibrium phase evolution in quantum materials.^1^ Most prior pump–probe studies have relied on transient reflectance or transmittance measurements, which in many cases are limited to single-wavelength probing and therefore provide only partial access to the underlying ultrafast dynamics.^2–7^ Even so, conventional transient reflectivity measurements can be highly sensitive to both photoinduced electronic relaxation and coherent lattice dynamics, including coherent acoustic phonons (CAPs). Their central limitation, however, is that they offer only indirect access to the microscopic optical response: the measured signal reflects a combined contribution from changes in both the dispersive and absorptive optical properties, making it difficult to disentangle the two.

Time-resolved pump–probe spectroscopic ellipsometry (TRSE) has recently advanced ultrafast optical probing by providing direct and precise access to the real and imaginary components of the nonequilibrium, time-dependent dielectric response ε­(ω,*t*).^8^ By tracking the amplitude ratio (ψ) and phase difference (Δ) of p- and s-polarized reflected light with femtosecond temporal resolution and broadband spectral coverage, TRSE directly yields the full complex dielectric function without recourse to Kramers–Kronig analysis, providing enhanced sensitivity to electronic^9–11^ and lattice dynamics.^12–14^ Unlike conventional transient reflectance or transmittance measurements, which typically reduce the photoinduced response to a single scalar observable, TRSE directly resolves the transient real and imaginary components of the dielectric function, ε_1_(ω,t) and ε_2_(ω,t), and thus photoinduced changes in optical dispersion and absorption. This enables photoinduced changes in optical dispersion and absorption to be distinguished, providing independent constraints on the microscopic origin and character of the underlying ultrafast dynamics.

This capability has enabled detailed investigations of carrier relaxation,^9,11,15^ entangled electron–phonon states,^14^ and coherent phonon excitation^12,13^ across a wide range of semiconductors, as well as ultrafast metal-to-insulator transitions (MITs) in correlated oxides such as VO_2_ and LaCoO_3_.^12,16,17^ TRSE is also sensitive to coherent acoustic phonons (CAPs),^12,13^ which are generated through photoinduced stress and propagate as coherent strain waves that dynamically modulate the dielectric response. Their oscillatory signatures can therefore provide information on strain generation and propagation, sound velocity, elastic properties, and electron–phonon coupling under nonequilibrium conditions.^18,19^ Importantly, resolving these CAP-induced modulations in the complex dielectric function enables their effects on^20^ both the dispersive and absorptive optical response to be distinguished.

Rare-earth nickelates (*RE*NiO_3_, where *RE* represents a rare-earth cation) are a class of strongly correlated quantum materials that exhibit metal–insulator transition, paramagnetic-antiferromagnetic transition, charge ordering, and orbital ordering, making them interesting candidates for both basic science^21^ and emerging applications in high-speed computing and communications.^22^ Among this family, NdNiO_3_ has emerged as a prototypical nickelate that is metallic at room temperature. Understanding the room-temperature evolution of its properties at femtosecond time scales upon photoexcitation is of broad interest in ultrafast and tunable device technologies that incorporate quantum materials. In particular, the transient complex dielectric function directly determines technologically relevant optical parameters, such as the refractive index, extinction coefficient, optical losses, and phase response, which ultimately govern the operation of active photonic devices. Consequently, quantifying the ultrafast evolution of the dielectric function is important both for understanding the nonequilibrium optical response of NdNiO_3_ and for assessing its potential as a dynamically tunable optical material.

Previous ultrafast studies of NdNiO_3_ have predominantly investigated photoinduced insulator-to-metal transitions in low-temperature insulating states using transient reflectivity or conductivity measurements.^2–7^ In contrast, the ultrafast evolution of the complex dielectric function under room-temperature conditions remains comparatively unexplored. Direct access to the transient complex dielectric function and its relation to coherent lattice excitations remains limited despite being critical for establishing how ultrafast electronic and structural dynamics modify the fundamental optical properties of these materials under nonequilibrium conditions.

Here, we employ broadband time-resolved spectroscopic ellipsometry to investigate the ultrafast optical response of a NdNiO_3_ thin film at room temperature following femtosecond photoexcitation. By directly tracking the temporal evolution of the complex dielectric function with sub-picosecond resolution, we quantitatively determine how these processes modify the dispersive and absorptive components of the optical response. In addition, we observe pronounced coherent acoustic phonons, whose signatures in the dielectric function provide insight into ultrafast strain generation and lattice dynamics in this correlated oxide system.

## Results and Discussion

A 50-nm-thick NdNiO_3_ (NNO) film was grown on an LaAlO_3_ (LAO) substrate using a sputtering system (see the Materials and Methodssection for details). The broadband transient pseudodielectric function Δ⟨ϵ⟩ of the NNO thin film on the LAO substrate was probed in the 318 to 729 nm range using femtosecond pump pulses at three different pump wavelengths: λ_pump_ = 266 nm (4.66 eV), λ_pump_ = 400 nm (3.1 eV), and λ_pump_ = 800 nm (1.55 eV). The pseudodielectric function ⟨ϵ⟩ measured by TRSE represents a dielectric function obtained directly from the measured ellipsometric parameters (Ψ, Δ) and is calculated from an optical model that assumes a semi-infinite flat substrate. Because the LAO substrate is known not to exhibit a photoinduced response under the pump conditions used in this study, any transient changes observed in the pseudodielectric function can be attributed entirely to the NNO film rather than the substrate. This makes the pseudodielectric function useful for identifying changes in the NNO properties driven by ultrashort laser pulses. The complex dielectric function ϵ = ϵ_1_ + *i*ϵ_2_ of the thin film at different time delays after any of the studied pumps can be derived by fitting to a multilayer model as the one used to extract the dielectric function of the static NNO film and shown in Table S1.

For all excitation conditions, comparable pump irradiances were employed to ensure that the observed variations in the transient optical response primarily originate from the excitation wavelength rather than differences in pump irradiances. The pump irradiances used for the different λ_pump_ were *I*_266 nm_ = 19.6 W/cm^2^, *I*_400 nm_ = 21.8 W/cm^2^, and *I*_800 nm_ = 21.7 W/cm^2^. Although the applied irradiances are nearly identical, the photon energy scales inversely with wavelength (*E* = *hc*/λ), meaning that similar irradiances correspond to different incident photon densities. As a result, longer-wavelength excitation, such as 800 nm, injects a larger number of photons into the sample than shorter-wavelength excitation. This difference in photon flux may lead to distinct excitation pathways and should therefore be considered when interpreting the wavelength-dependent ultrafast dynamics.

Figure 1a–c shows the transient pseudodielectric function Δ⟨ϵ⟩ of the NNO film as a function of pump–probe delay time and wavelength for the different λ_pump_. Similar features in Δ⟨ϵ⟩ are observed regardless of λ_pump_. Two distinct dynamic regimes can be identified, as exemplified in Figure 2 for λ_pump_ = 266 nm. First, within the first picosecond after excitation, a sharp increase and decrease in Δ⟨ϵ⟩ is observed (highlighted in yellow in Figure 2c), accompanied by oscillations in the photon energy domain. Figure 2b shows these oscillations measured 100 fs after the pump. The characteristic lifetime is on the order of a few hundred femtoseconds, as will be discussed in more detail in Figure 4. Second, in the 5–100 ps range, a long-lived transient response emerges, characterized by oscillations in the time domain (highlighted in green in Figure 2c). To disentangle the different physical processes contributing to the overall optical response, we analyze these two dynamic regimes separately, focusing first on the sub-picosecond response and then on the slower dynamics in the 5–100 ps range.

**Figure 1:**
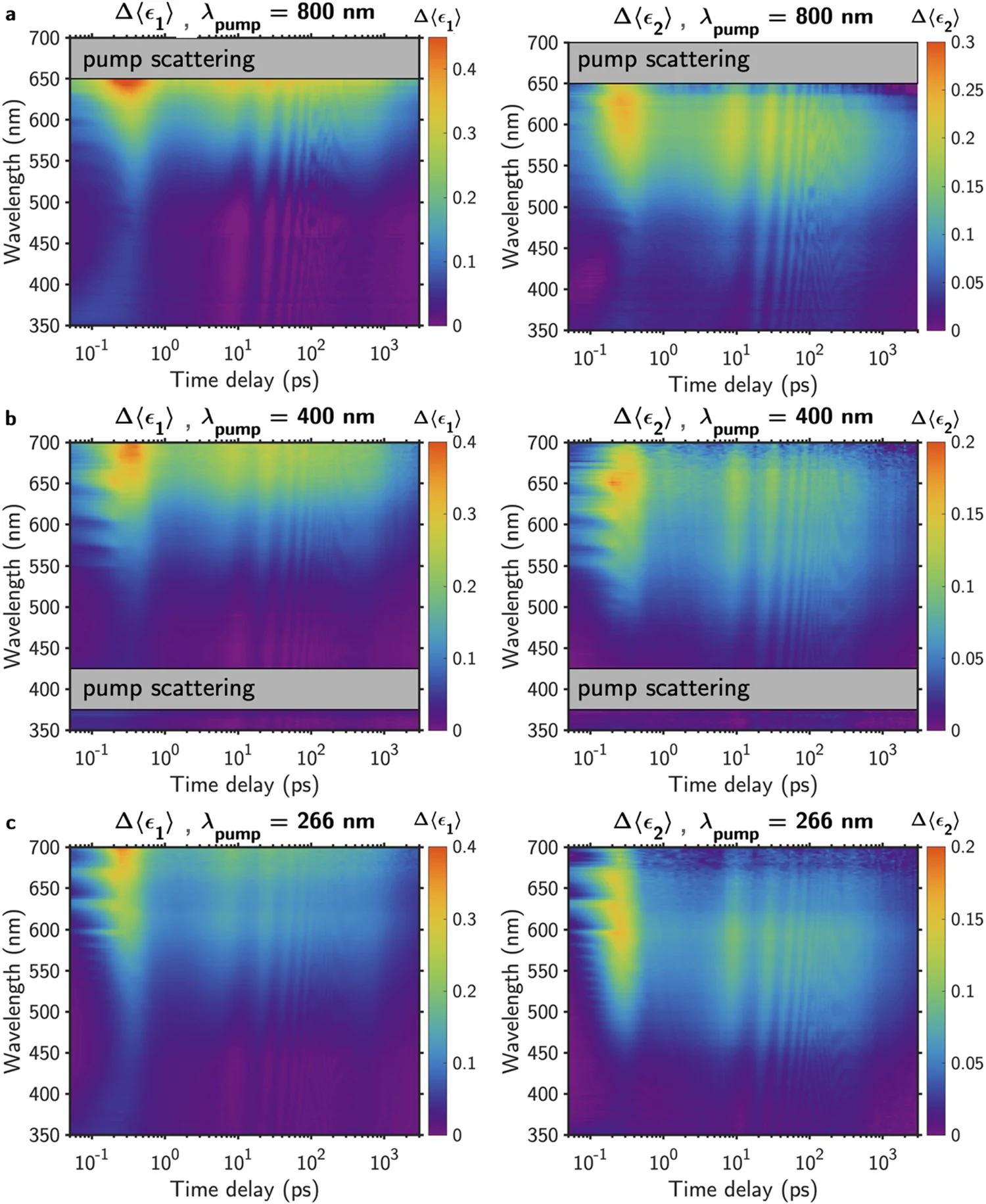
Transient real (left) and imaginary (right) parts of pseudodielectric function Δ⟨ϵ⟩ = Δ⟨ϵ_1_⟩ + *i*Δ⟨ϵ_2_⟩ as a function of the photon energy and time delay for pump wavelengths of (a) 800 nm, (b) 400 nm, and (c) 266 nm. The gray shaded regions indicate regions where pump scattering creates artifacts and have been excluded from the analysis.

**Figure 2:**
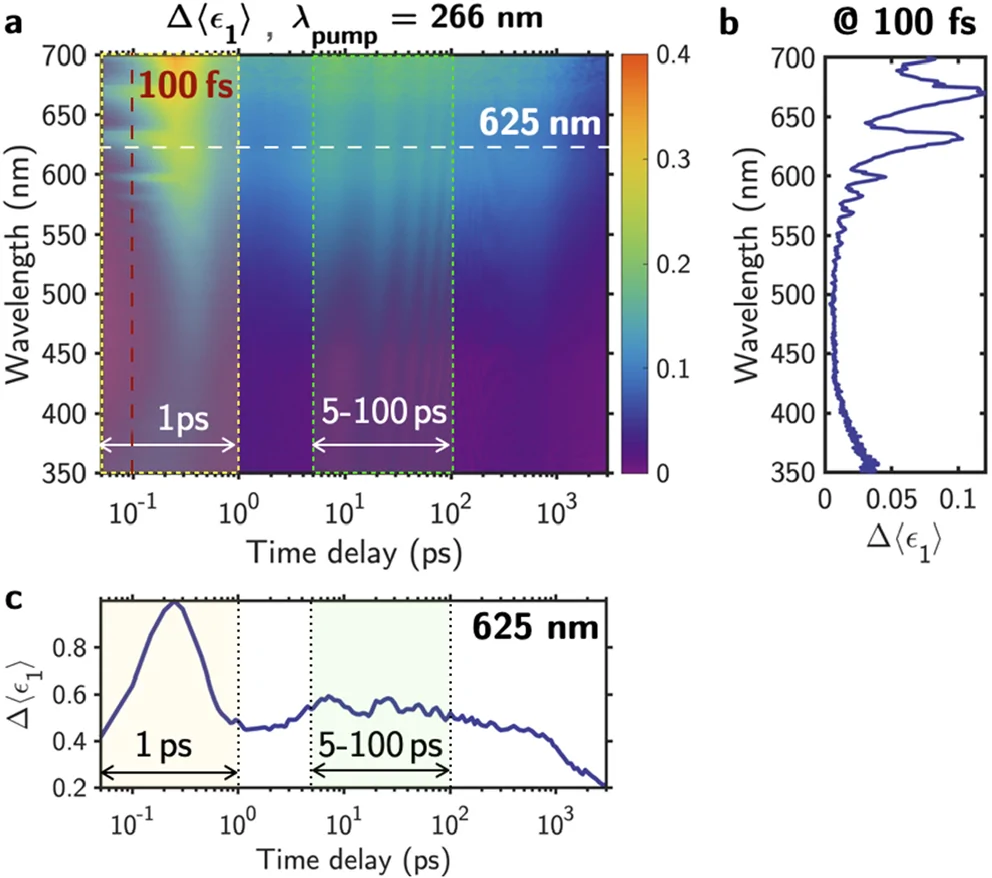
(a) Transient real part of pseudodielectric function Δ⟨ϵ_1_⟩ as a function of the photon energy and time delay for pump wavelength 266 nm. (b) Δ⟨ϵ_1_⟩ spectrum 100 fs after the pump showing oscillation in the spectral domain. (c) Δ⟨ϵ_1_⟩ as a function of the time delay probed at 625 nm showing a sharp increase and decrease within the first picosecond after the pump pulse, followed by oscillations in the time domain between 5 and 100 ps.

Figure 3a shows the normalized transient response as a function of time delay within the first 2 ps, monitored at 625 nm and for three different pump wavelengths. Immediately after excitation, the signal exhibits a sharp increase, reaching a maximum at 250 fs (266 nm), 350 fs (400 nm), and 450 fs (800 nm), with the peak occurring progressively later for longer wavelengths, followed by a decay toward a steady-state value higher than the static value. We attribute this rapid rise to changes in the electronic population resulting from the increased density of photoexcited electrons generated by the pump pulse.

**Figure 3:**
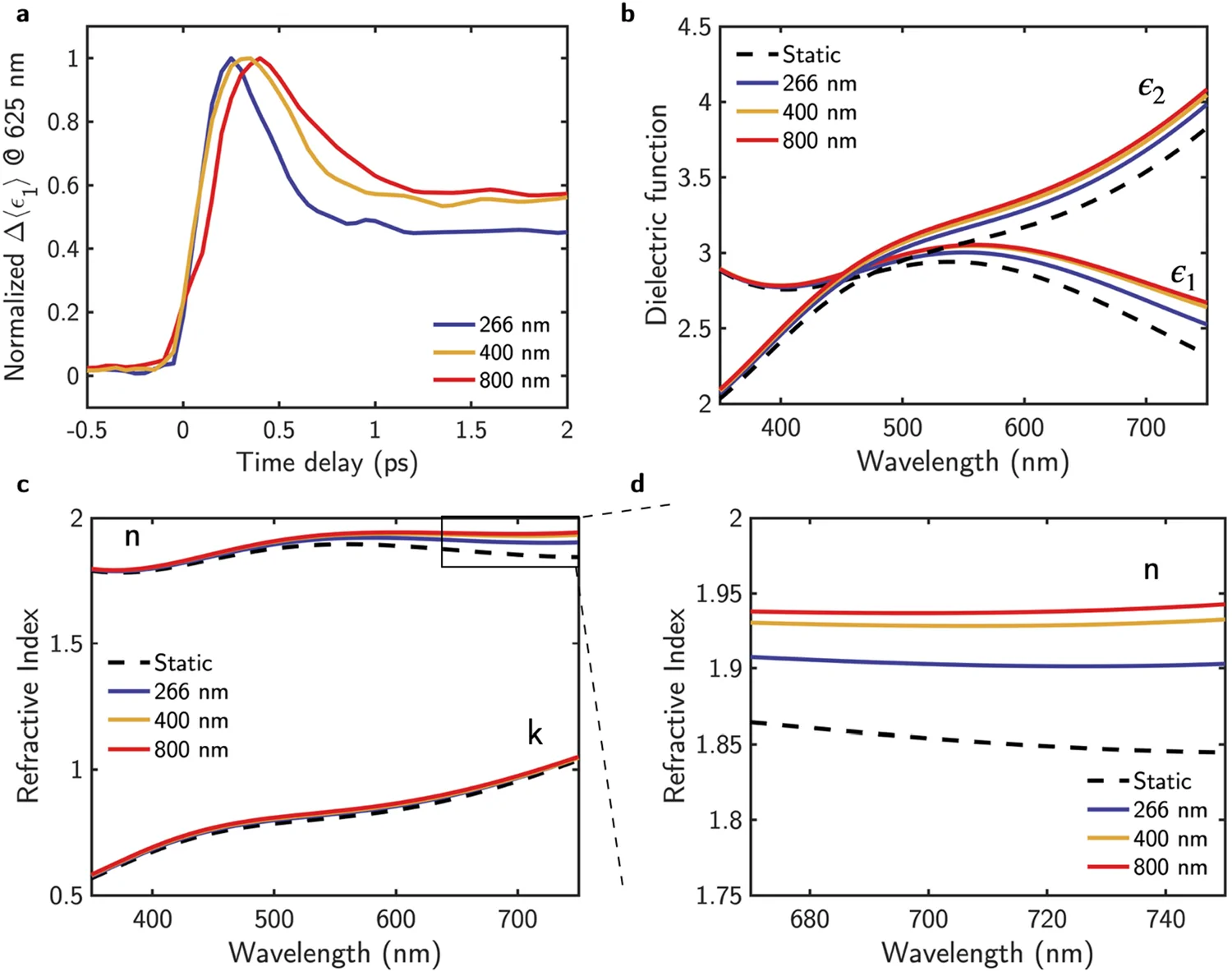
(a) Normalized values of Δ⟨ϵ_1_⟩ monitored in time at 625 nm in the first 2 ps after the pump. (b) Dielectric function (ϵ_1_ + *i*ϵ_2_), (c) complex refractive index (*n* + *ik*), and (d) zoomed refractive index variation of the film, all shown for before the pump (static) and at peak pump-induced response: 250 fs (λ_pump_ = 266 nm), 350 fs (λ_pump_ = 400 nm), and 450 fs (λ_pump_ = 800 nm).

Figure 3b displays the extracted transient dielectric function (ϵ_1_ + *i*ϵ_2_) of NNO before excitation (static) and at the delay corresponding to the maximum pump-induced response, extracted from the time-resolved ellipsometric spectra (Ψ and Δ). Figure 3c,d shows the corresponding complex refractive index (*n* + *ik*) and a zoomed view of its variation, respectively. At these peak delays, the optical constants exhibit their largest deviation from the static state. One Drude and three Lorentz oscillators were used to fit the ellipsometric spectra, as shown in Table 1. For all three pump wavelengths, both the dielectric function and refractive index exhibit the same overall trend, consistent with the nearly zero room-temperature bandgap of NNO,^23^ which leads to similar excitation dynamics regardless of pump photon energy.

**Table 1:** Ellipsometry Fitting Model of the Undoped NNO before and after the Pump Excitation

pump type	Drude	Lorentz 1	Lorentz 2	Lorentz 3
static	*A* = 8.342 eV, *B*_r_ = 0.885 eV	*A*_1_ = 4.098 eV	*A*_2_ = 1.774 eV	*A*_3_ = 2.985 eV
266 nm	*A* = 6.762 eV, *B*_r_ = 1.113 eV	*E*_n1_ = 7.094 eV	*E*_n2_ = 2.777 eV	*E*_n3_ = 1.150 eV
400 nm	*A* = 6.146 eV, *B*_r_ = 1.242 eV	*B*_r1_ = 3.315 eV	*B*_r2_ = 2.543 eV	*B*_r3_ = 1.033 eV
800 nm	*A* = 5.973 eV, *B*_r_ = 1.299 eV			

To better understand the pump-induced changes in the ellipsometric model, we modeled the transient response by allowing only the Drude term to vary while keeping the three Lorentz oscillators fixed since photoexcitation primarily modifies free carrier properties in this metallic system. The fits show good agreement with the measured experimental data. As shown in Table 1, photoexcitation reduces the Drude amplitude *A* while increasing the broadening parameter *B*_r_ across all pump wavelengths, indicating suppression of the Drude response together with enhanced carrier scattering, respectively. Although increasing the pump wavelength increases the incident photon flux, Drude amplitude *A* decreases progressively. This indicates that the additional photoexcited carriers do not enhance the free carrier response. In strongly correlated nickelates, photoexcited carriers do not necessarily contribute to a delocalized Drude response but may instead undergo transient localization and/or redistribution of the Drude spectral weight to incoherent finite-energy excitations.^24–26^ This observation is further supported by the simultaneous increase in the Drude broadening parameter *B*_r_, which independently indicates enhanced carrier scattering and a decrease in the carrier lifetime.

When examining this ultrafast response in the photon energy domain, we observe the emergence of a pronounced periodic spectral modulation in Δ⟨ϵ_1_⟩ during the first 200 fs. Figure 4a–d displays the Δ⟨ϵ_1_⟩ spectra within this time window for each λ_pump_. These oscillations are clearly visible for all the λ_pump_; a close-up view of the ones appearing for λ_pump_ = 266 nm is shown in Figure 4b. To quantify the characteristic periodicity of these spectral features, we performed a fast Fourier transform (FFT) along the photon energy axis. We emphasize that this analysis does not imply an energy-time correspondence and is not used to extract a dynamical time scale; rather, it provides a measure of the dominant spectral periodicity present in the transient dielectric response. FFT analysis reveals dominant frequencies of ≈ 54 meV (436 cm^–1^) for λ_pump_ = 266 nm and ≈ 120 meV (967 cm^–1^) for λ_pump_= 400 and 800 nm.

**Figure 4:**
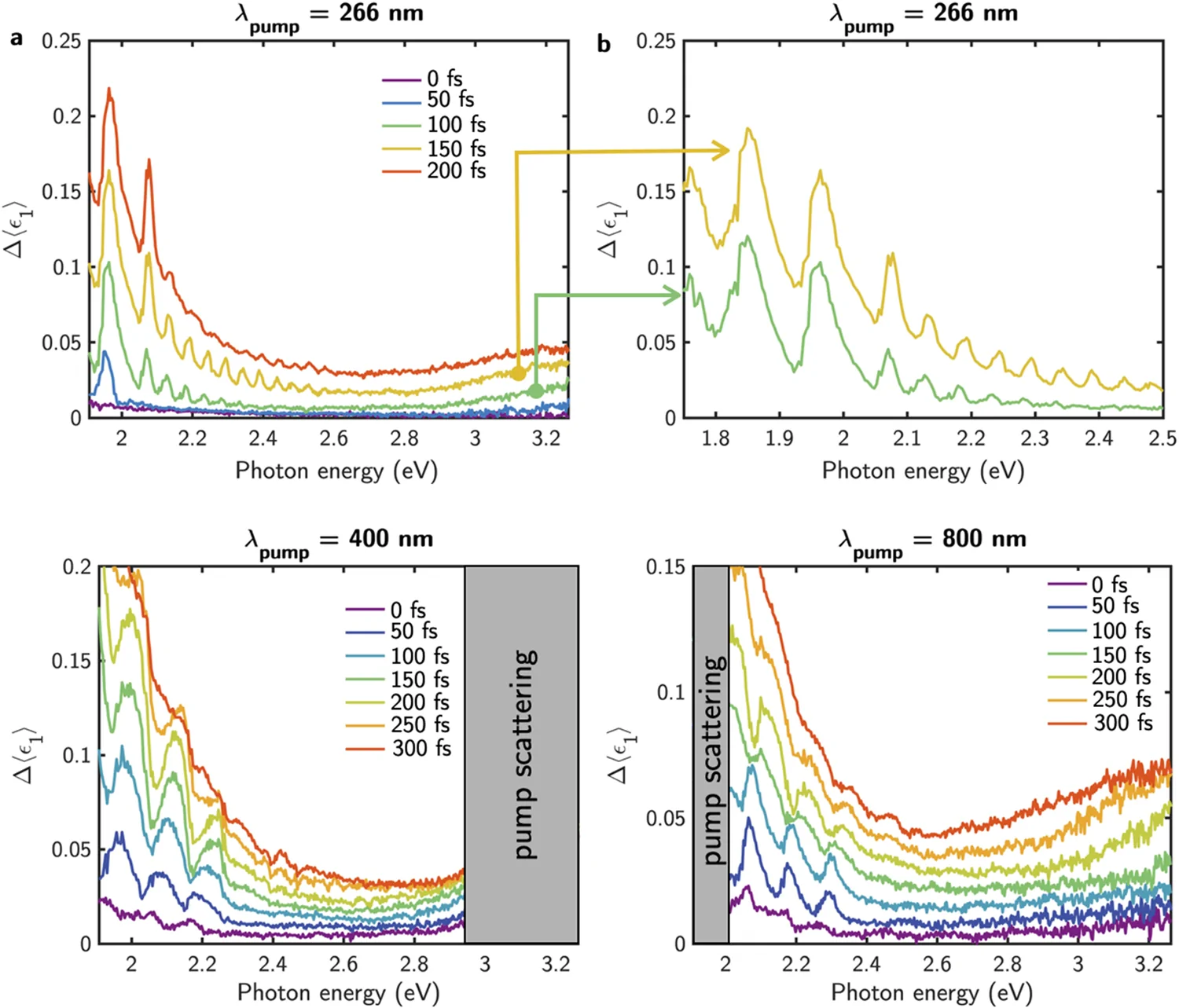
Δ⟨ϵ_1_⟩ spectra in the first 200 fs after the pump for the pump wavelengths (a) 266 nm, (c) 400 nm, and (d) 800 nm. (b) Close-up view of the oscillatory features observed at 100 and 150 fs after excitation with the 266 nm pump. The gray shaded regions indicate regions where pump scattering creates artifacts and have been excluded from the analysis.

The spectral periodicity of 436 cm^–1^ is consistent with the frequency of Ni–O bending and stretching modes of the NiO_6_ octahedra reported for NNO,^27^ supporting its assignment to coherent optical phonons generated during the initial stages of carrier relaxation. Importantly, this interpretation is not based solely on the Fourier analysis, but also on the correspondence between the extracted energy periodicity and known optical phonon modes of NdNiO_3_,^28^ as well as on the close similarity to the energy-dependent dielectric function modulations reported for photoexcited Si and attributed to coherent optical phonons.^14^ The higher frequency observed under 400 nm excitation (967 cm^–1^) does not correspond to any known zone-center phonon of NNO, and may reflect a multiphonon process, an overtone activated by the higher photon energy, or alternatively, quantum beats arising from the coherent superposition of photoexcited electronic states with closely spaced energies, a phenomenon well documented in pump–probe spectroscopy of correlated oxides and solid-state systems on similar time scales.^29,30^ The amplitude of these spectral modulations decreases rapidly, vanishing within approximately 200 fs for λ_pump_ = 266 nm and 300 fs for λ_pump_= 400 and 800 nm, consistent with rapid dephasing driven by electron–electron and electron–phonon scattering,^14^ which is the expected time scale for electronic decoherence. The surviving incoherent optical phonon population subsequently decays into lower-energy acoustic phonons via anharmonic phonon–phonon scattering.

Finally, we turn our attention to the long-lived oscillations observed in the time domain between 10 and 100 ps. These oscillations originate from a coherent acoustic strain pulse generated in the NNO film following photoexcitation, and their detection provides an indirect but quantitative signature of the photoinduced lattice response. Because LAO is essentially transparent at the pump wavelengths employed in this work, optical excitation and the associated photoinduced stress are predominantly confined to the NNO layer. However, the time window investigated here corresponds to delay times comparable to or longer than the acoustic transit time across the 50-nm-thick film. Consequently, the strain pulse crossed the NNO/LAO interface and propagated into the substrate. Because of the relatively small acoustic impedance mismatch between NNO and LAO, a substantial fraction of the acoustic pulse is transmitted into the substrate. The CAPs are therefore generated within the NNO film, but it is their propagation within LAO that is actually detected, manifested as the Brillouin oscillations discussed below. In this sense, the substrate acts as a well-characterized acoustic ″detector″ for the strain pulse launched by the photoexcited film, complementing the electronic signatures of NNO discussed in the preceding sections.

Figure 5a displays these oscillations in Δ⟨ϵ_1_⟩, monitored at 625 nm, for the different λ_pump_ used in the experiment. The temporal response between 10 and 100 ps exhibits clear oscillatory behavior, which can be modeled using a damped harmonic oscillator Δ⟨ϵ_1_⟩(*t*) = *A* + *B*sin­(2π*ft* + ϕ)*e* ^–*αt*^, characteristic of Brillouin oscillations.^28^ The oscillatory contribution was isolated from the nonoscillatory electronic and thermal relaxation background of the NNO through nonlinear fitting to the damped sinusoidal response, from which the oscillation frequency *f* and period τ = *f* ^–1^ extracted at each probe wavelength together with their uncertainties via nonlinear least-squares fitting with a Jacobian-based 95% confidence interval.

**Figure 5:**
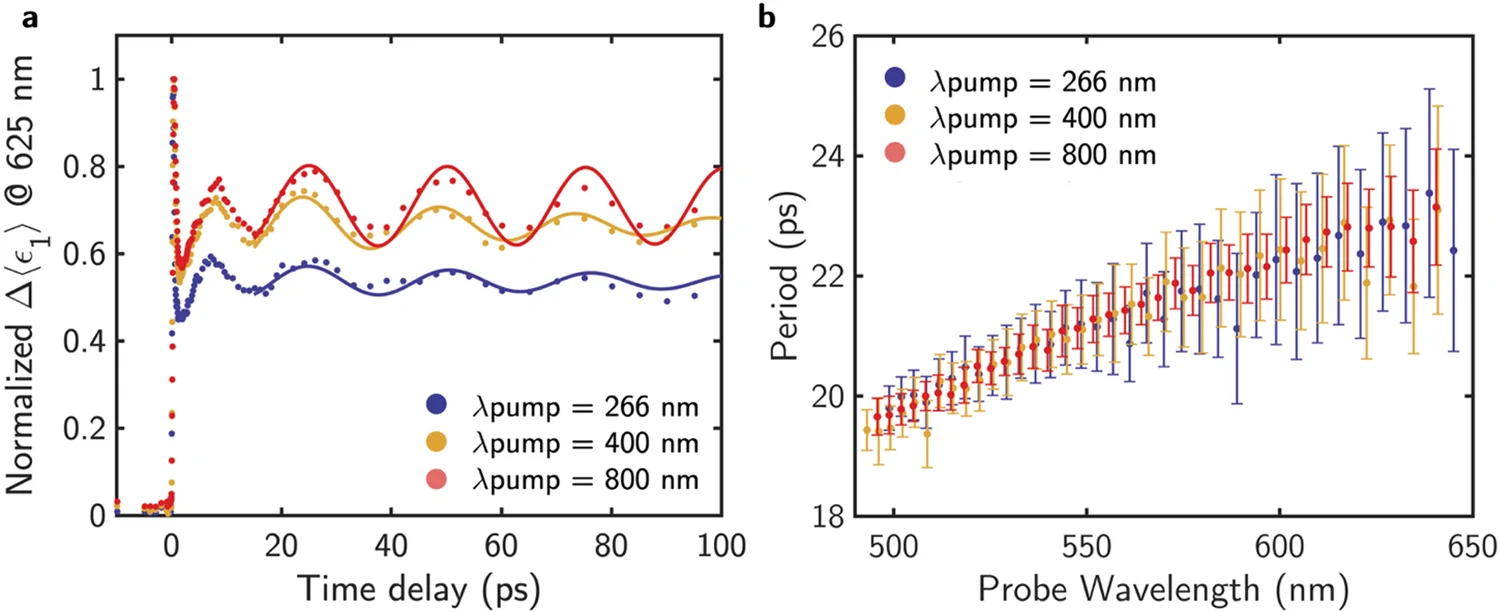
(a) Normalized values of Δ⟨ϵ_1_⟩ monitored in time at 625 nm in the first 100 ps after the pump. (b) Brillouin oscillation period as a function of probe wavelength for the three pump wavelengths. Error bars represent 95% confidence intervals from the nonlinear least-squares fits. The wavelength dependence of the oscillation period is consistent with a time-domain Brillouin scattering process associated with the propagation of the photoinduced acoustic strain pulse into the substrate.

After traversing the NNO film and propagating into the LAO substrate, the coherent acoustic strain pulse launched by NNO’s photoinduced stress gives rise to Brillouin oscillations in the transient ellipsometry signal, appearing as a periodic modulation of Δ⟨ϵ_1_⟩ as a function of time delay. These oscillations arise from the interference between probe light reflected at the film surface and light scattered by the moving strain pulse front, and their period τ is directly linked to the longitudinal sound velocity *v* of the substrate through the relation τ = λ/(2*vn* cos β), where λ is the probe wavelength, *n* = 2.05 is the real part of the refractive index of LAO,^31^ and $\cos\beta =\sqrt{1‐\sin^{2}\alpha /n^{2}}$ is the cosine of the refraction angle inside the film, with AOI = 65° being the angle of incidence of the probe beam.^32^ Because this relation depends only on well-established optical and elastic constants of LAO, it offers a self-consistency check on the strain-pulse picture: if the oscillations detected at each wavelength are indeed governed by the propagation of the NNO-launched pulse through a homogeneous LAO medium, the extracted *v* should be wavelength- and pump-independent and should agree with the known bulk value. To extract the oscillation period at each probe wavelength, the transient Δ⟨ϵ_1_⟩(*t*) signal in the 10–100 ps window was fitted to a damped sinusoidal function from which the oscillation frequency *f* and hence the period τ are obtained at each wavelength together with their uncertainties. The sound velocity at each probe wavelength is then computed as *v* = λ/(2τ*n* cos β), and a weighted mean is taken across all probe wavelengths, with weights given by the inverse squared uncertainty of each estimate. This point-by-point analysis was adopted instead of extracting *v* from the slope of a linear fit of τ versus λ, since the accessible spectral range (500–650 nm) is relatively narrow and therefore insufficient for a robust slope determination. In addition, free-intercept linear fits are particularly sensitive to systematic offsets in τ arising from background subtraction and from the weak spectral dispersion of *n*.

The resulting Brillouin oscillation period as a function of probe wavelength is shown in Figure 5b for all three pump conditions. Applying this procedure independently for each pump wavelength yields *v* = 7.00 ± 0.04, 7.03 ± 0.03, and 7.04 ± 0.02 nm/ps for λ_pump_ = 266, 400, and 800 nm, respectively, all consistent within experimental uncertainty. The absence of a measurable dependence on pump wavelength confirms that, once launched, the acoustic pulse propagates as a coherent wavefront whose detected period is governed by the elastic and optical properties of the LAO substrate rather than by the specific optical excitation pathway used to generate it in NNO, precisely as expected if the substrate is acting as a passive but faithful carrier of the photoinduced strain. Taking the weighted mean across all pump conditions, we obtain a longitudinal sound velocity of *v* = 7.03 ± 0.02 nm/ps, in good agreement with previously reported longitudinal acoustic velocities in LAO. Because our measurement geometry does not align along a single crystallographic direction, the extracted value should be regarded as an effective sound velocity averaged over the orientations sampled by the probed acoustic wavevectors, rather than a velocity along a specific crystal axis. In particular, room-temperature measurements have reported values ranging from 6.72 nm/ps along [100] to approximately 7.9 nm/ps along [110] and [111],^33^ reflecting the crystallographic anisotropy of the elastic properties of LAO. More recent time-resolved optical measurements reported 6.67 nm/ps along [001].^34^ Our value falls within this range of literature-reported directional velocities, consistent with an orientationally averaged response. This agreement validates the strain-pulse interpretation of the observed oscillations and establishes transient ellipsometry as a reliable probe of the coherent acoustic response photoinduced in NNO, supporting its use as a diagnostic tool for photoinduced strain dynamics in complex oxide heterostructures more broadly.

## Conclusions

We have employed broadband time-resolved spectroscopic ellipsometry to investigate the ultrafast optical response of the NNO thin film at room temperature under femtosecond photoexcitation at 266, 400, and 800 nm. The transient dielectric function reveals two well-separated dynamic regimes. In the sub-picosecond regime, photoexcitation produces a rapid increase in the transient pseudodielectric function attributed to nonequilibrium carrier generation, accompanied by a reduction in Drude spectral weight and increased carrier scattering across all pump wavelengths. This behavior is indicative of a transient redistribution of the spectral weight and enhanced carrier scattering. Coherent optical phonon oscillations observed within the first 200 fs decay rapidly because of electronic decoherence.

At longer time scales, coherent acoustic strain manifests as Brillouin oscillations in the transient ellipsometric signal between 10 and 100 ps. Considering the 50 nm thickness of the NdNiO_3_ film, the acoustic pulse traverses the film within approximately 10 ps and subsequently propagates into the substrate. The long-time oscillations are therefore assigned to time-domain Brillouin scattering associated with the propagating strain pulse in the LAO substrate. These observations demonstrate that photoexcitation generates coherent acoustic strain that persists beyond the film transit time and modulates the measured optical response. The overall physical picture emerging from the present measurements is summarized schematically in Figure 6.

**Figure 6:**
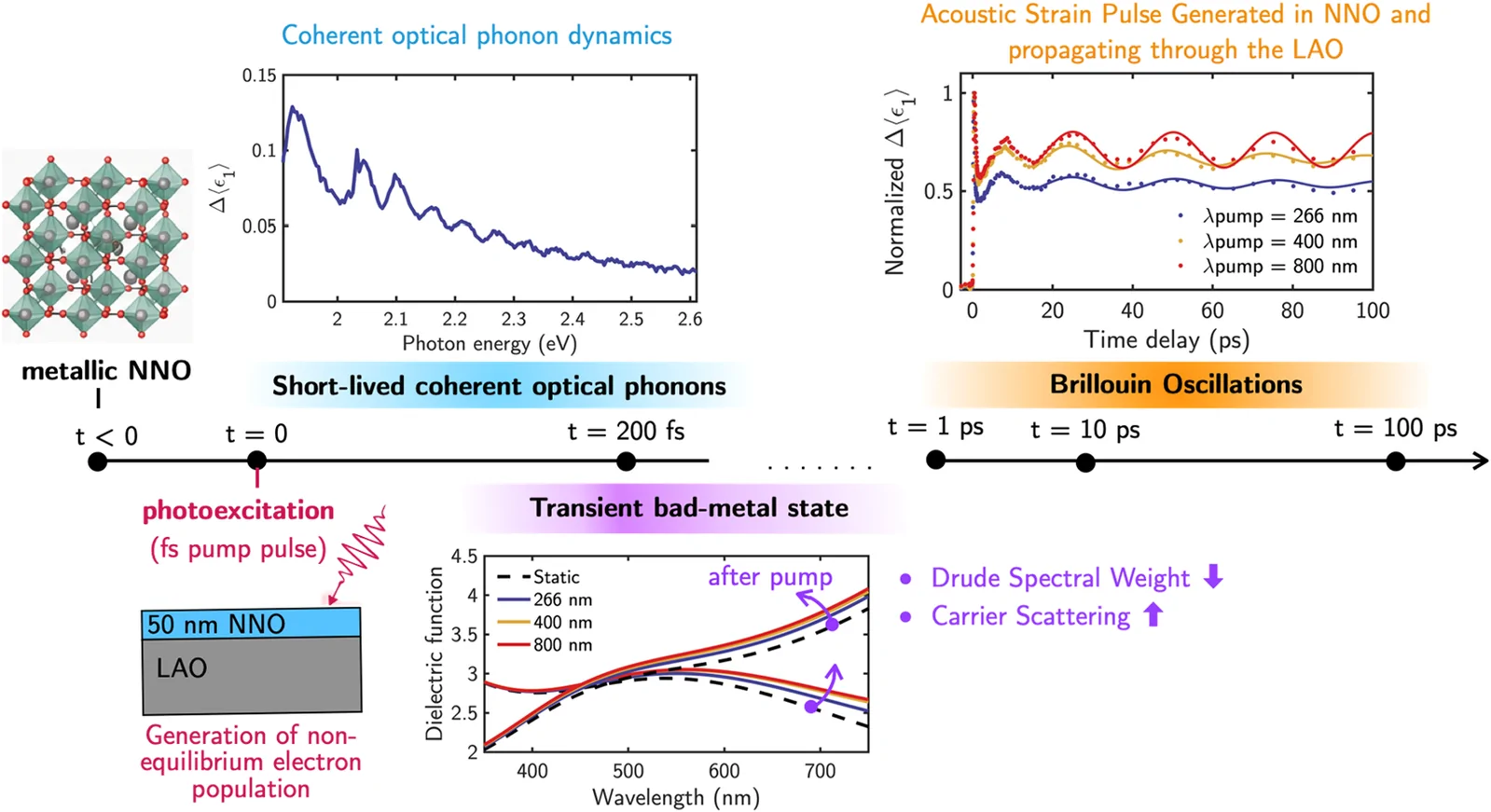
Schematic summary of the ultrafast dynamics in NdNiO_3_ revealed by broadband time-resolved spectroscopic ellipsometry.

## Materials and Methods

### Thin Film Preparation and Characterization

A 50-nm-thick NNO film was deposited on single-crystal (100)-oriented LaAlO_3_ (LAO) substrates using a radiofrequency (RF) magnetron sputtering system with high-purity Nd and Ni targets. During growth, an RF power of 135 W was applied to the Nd target and a DC power of 75 W to the Ni target. The deposition was performed at room temperature under a pressure of 5 mTorr, maintained by flowing 30 SCCM of Ar and 10 SCCM of O_2_ gases. The substrate holder was rotated at 40 rpm to ensure uniform film thickness. Postdeposition, the films were annealed in air at 500 °C for 24 h in a tube furnace.

Figure S1a shows the X-ray diffraction (XRD) patterns of the NNO films and of the bare LAO substrate, measured around the (002)_pc_ reflection (pc denotes the pseudocubic setting) using a Panalytical X’Pert diffractometer with Cu Kα radiation (λ = 1.5406 Å). The data taken on the NNO film exhibits sharp substrate peak and a film peak (marked with *), confirming the crystalline phase formation. The out-of-plane lattice constant (*c*_pc_) of the NNO film was calculated to be 3.824 Å, consistent with literature values.^35,36^

To estimate the elemental composition, Rutherford Backscattering Spectroscopy (RBS) of the NNO film was performed using a 1.7-MV IONEX tandem accelerator, which utilized a 2.3-MeV He^2+^ ion beam with a 2 mm diameter. The detector was set at a scattering angle of 163° with a resolution of 18 keV. Data analysis for the RBS results was conducted using the SIMNRA program.^37^ Figure S1b shows the experimental and simulated RBS spectra of the NNO film. The spatially averaged areal density ratio of Nd:Ni:O was found to be approximately 0.21:0.19:0.6, confirming the stoichiometry of the NNO film.

The electrical transport properties of the NNO film were characterized by measuring its resistance as a function of temperature between 300 and 400 K (Figure S1c) using an INSTEC HP1000V-PM probe station with Keithley 2635A source meter. The monotonic increase in the resistance with increasing temperature is characteristic of metallic conduction.

The optical properties of the NNO film grown on LAO were first characterized by conventional spectroscopic ellipsometry. Ellipsometric spectra were acquired at an incidence angle of 65° over the 250–1200 nm spectral range using a Park Systems EP4 ellipsometer. The experimental data were fitted using a dielectric function model consisting of one Drude term, supporting the metallic character of the film, and three Lorentz oscillators, with the extracted fitting parameters summarized in Table S1.^23^ This fitting procedure enabled the determination of both the film thickness and the complex refractive index. The NNO film thickness was determined to be 50 nm, and the extracted complex refractive index, shown in Figure S1d, is in good agreement with previously reported literature values.^23^

### Time-Resolved Pump–Probe Spectroscopic Ellipsometry

Time-resolved pump–probe spectroscopic ellipsometry (TRSE) was employed to monitor the temporal evolution of the complex pseudodielectric function following photoexcitation. The experimental setup was based on a broadband femtosecond ellipsometer operating in a polarizer-sample-compensator-analyzer (PSCA) configuration and its schematized in Figure S2. The light source was a femtosecond Coherent Astrella laser system delivering pulses at a central wavelength of 800 nm. A supercontinuum white-light probe (SCG in Figure S2) was generated by focusing the fundamental pulse into a CaF_2_ window, followed by spectral filtering using a Thorlabs FGS900-A bandpass filter. The resulting broadband probe, spanning from 1.7 to 3.9 eV (729 to 318 nm), was incident on the sample at 65°, enabling spectral measurements over a wide energy range.

Excitation was performed using the fundamental wavelength of 800 nm pump pulses, and wavelengths of 400 and 266 nm produced via second and third harmonic generation (SHG and THG). The pump beam was p-polarized using a half-wave plate and directed onto the sample at an angle of incidence of 60°, closely aligned with the probe beam to ensure near-collinear geometry ensuring spatial overlap so that the entire probe region was uniformly excited by the pump pulse. To allow full thermal relaxation between successive excitations, the pump repetition rate was set to 250 Hz. The spatial overlap of the beams was optimized such that the pump spot was larger than the probe, ensuring uniform excitation within the probed region. A mechanical delay stage (DL) in the pump path provided temporal resolution of the dynamic response. The pump pulse durations were approximately 35 fs, 50 fs, and 60 fs for 800, 400, and 266 nm, respectively. The pump–probe cross-correlation at the sample was approximately 100 fs for 400 nm and 110 fs for 266 nm. The probe and pump spot diameters were approximately 80 μm and >200 μm, respectively, with a 5° angle between the beams, resulting in a geometrical temporal spread of approximately 30 fs.

Transient reflectance-difference spectra were collected using a prism-based spectrometer coupled to a CCD camera operating at 1 kHz. Measurements were performed at multiple time delays and compensator angles. Background correction was carried out using three reference measurements: with only the pump, only the probe, and with both beams blocked. The extraction of the time-dependent pseudodielectric function involved Mueller matrix-based data analysis, chirp correction, and normalization to steady-state ellipsometric spectra (Ψ and Δ) acquired prior to the pump–probe experiment using a Park Systems EP4 ellipsometer. Additional details on the instrumentation and data processing protocol can be found in ref 8.^8^

## Supplementary Material



## Data Availability

The data underlying this study are openly available at Zenodo: (https://zenodo.org/records/20433965)

## Notes

Detailed characterization of the NNO films (XRD, RBS, temperature-dependent resistance and ellipsometry); ellipsometry fitting model of the NNO; and schematic of the experimental setup for the time-resolved pump–probe spectroscopic ellipsometry measurements (PDF)
