# Supplementary material for: Sub-Picosecond Optical Dynamics in Perovskite NdNiO3 Thin Films via Broadband Pump–Probe Ellipsometry
Source: ACS Photonics. 2026 Oct 6;13(20):6207–15. doi: 10.1021/acsphotonics.6c01634 (PMC13693500; doi:10.1021/acsphotonics.6c01634)
Supplement: Supplementary file 1 [file ph6c01634_si_001.pdf]

Supplementary Information for:

Sub-picosecond optical dynamics in perovskite $NdNiO_3$ thin films via broadband pump–probe ellipsometry

Yael Gutiérrez,[1] Ranjan Kumar Patel,[2] Saúl Vázquez-Miranda,[3] Mateusz Rebarz,[3] Shirly Espinoza,[3] Yeonghoon Jin,[4] Mikhail A. Kats,[4] Shriram Ramanathan[2]*

[1] Departamento de Física Aplicada, Universidad de Cantabria, Avenida de los Castros, s/n, 39005 Santander, Spain.

[2] Department of Electrical and Computer Engineering, Rutgers University, Piscataway, New Jersey 08854, USA.

[3] ELI Beamlines Facility, The Extreme Light Infrastructure ERIC, Za Radnicí 835, 25241 Dolní Břežany, Czech Republic.

[4] Department of Electrical and Computer Engineering, University of Wisconsin-Madison, Madison, WI 53706, USA

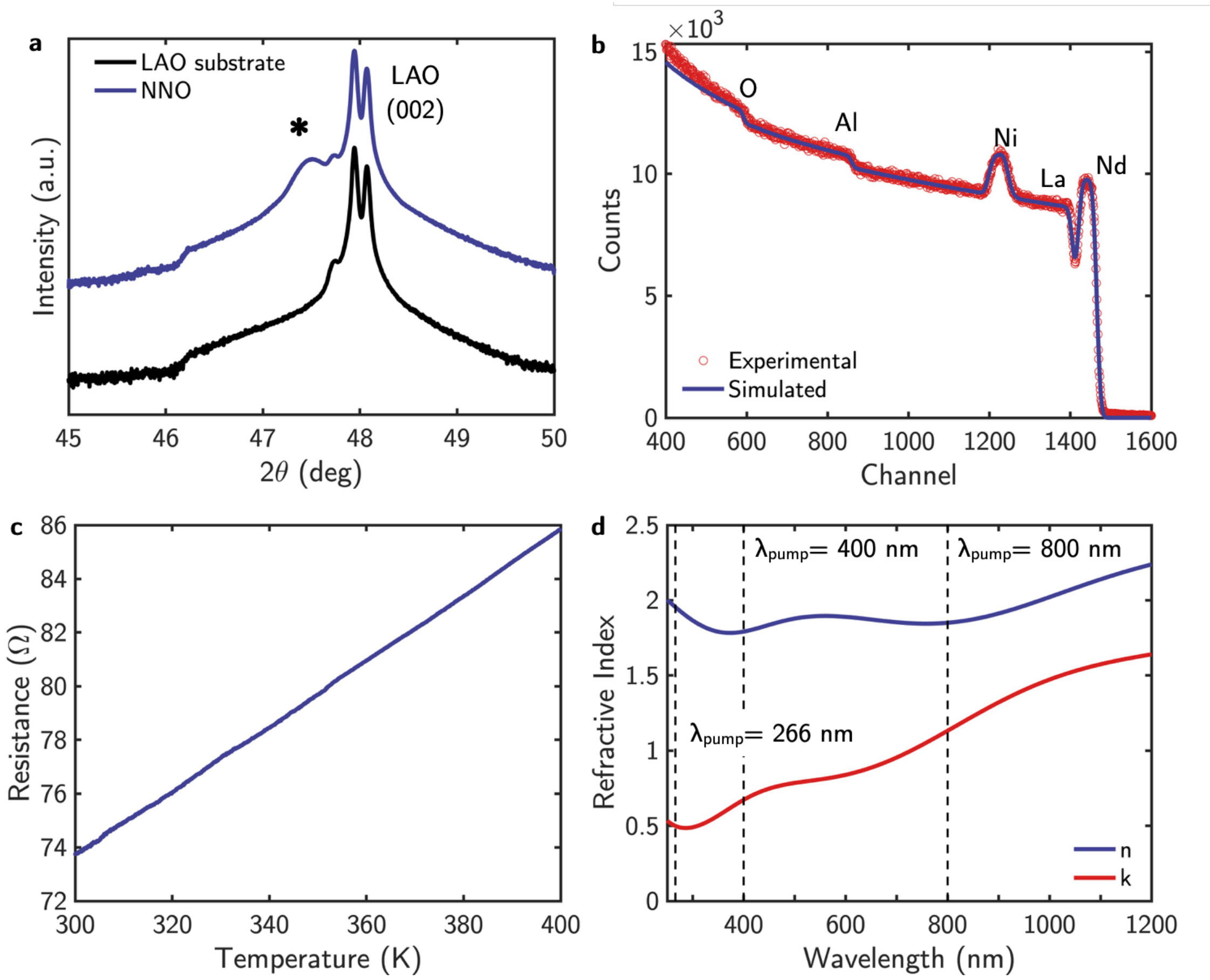

Figure S1. (a) XRD patterns of the NNO film, shown alongside the reference LAO substrate, measured around the (002) reflection. The NNO (002) film peak is marked with a symbol. (b) Experimental and simulated RBS spectra of the NNO film on a LAO substrate. (c) Temperature-dependent resistance of the NNO film. (d) Real and imaginary components of the complex refractive index $n + ik$ of a NNO film extracted from conventional spectroscopic ellipsometry measurements. The vertical lines indicate the wavelengths (266 nm, 400 nm and 800 nm) at which the optical pumping is performed in the pump-probe experiments.

Table S1. Ellipsometry fitting model of the NNO in Figure S1d.

Sample	ε_∞	Drude	Lorentz 1	Lorentz 2	Lorentz 3
NNO (50 nm)	1.977	$\boldsymbol{\varepsilon(E) = \varepsilon_\infty - \frac{A \cdot Br}{E^2 - iBrE}}$	$\boldsymbol{\varepsilon(E) = \varepsilon_\infty + \frac{A_n E_n}{E_n^2 - E^2 + iBr_n E}}$		
		A = 8.342 eV<br>Br = 0.885 eV	**A_1 = 4.098 eV**<br>**E_{n1} = 7.094 eV**<br>**Br_1 = 3.315 eV**	**A_2 = 1.774 eV**<br>**E_{n2} = 2.777 eV**<br>**Br_2 = 2.543 eV**	**A_3 = 2.985 eV**<br>**E_{n3} = 1.150 eV**<br>**Br_3 = 1.033 eV**

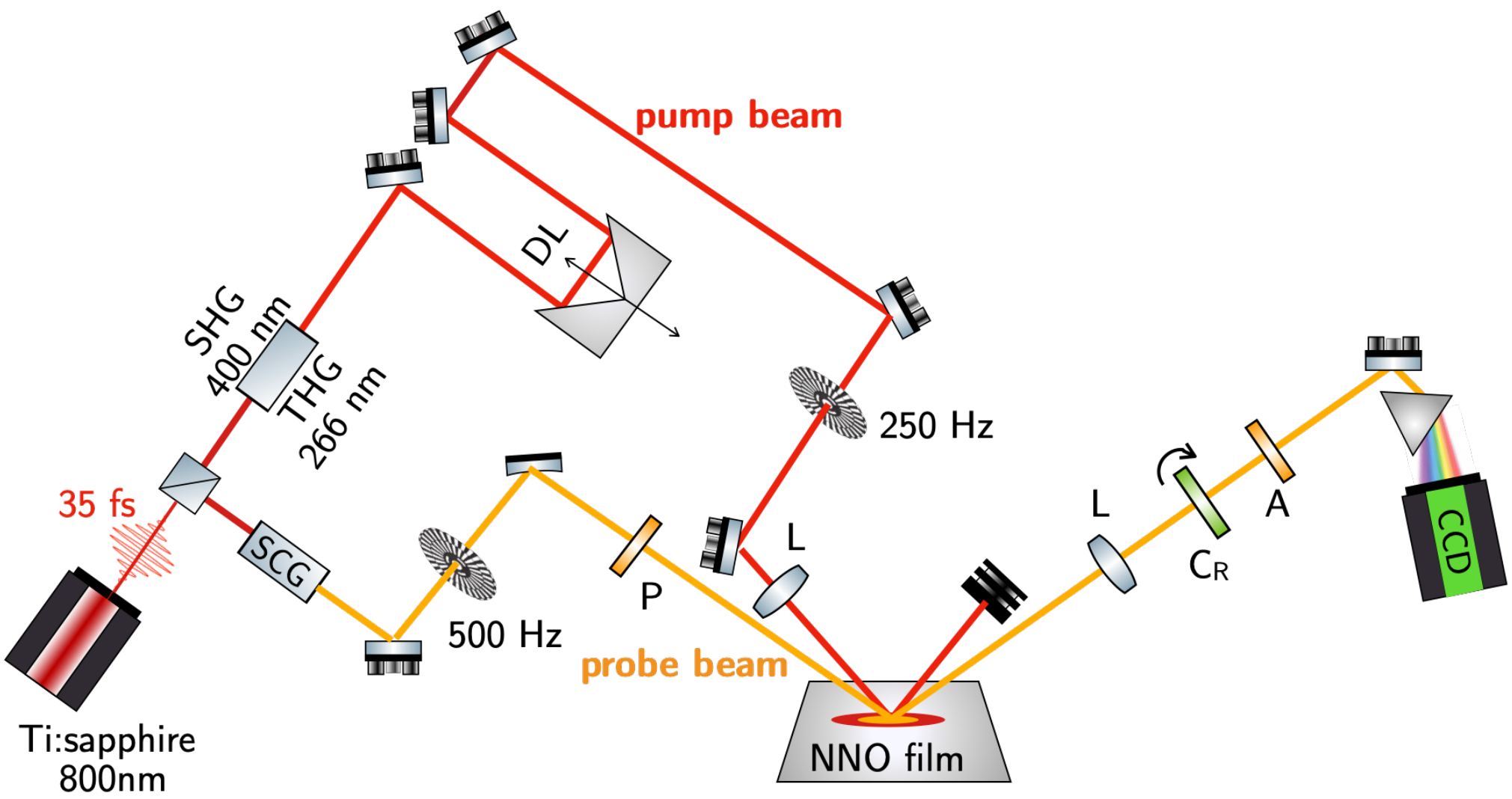

Figure S2. Schematic of the experimental set up for the time-resolved pump-probe spectroscopic ellipsometry measurements. SHG, second harmonic generation for the pump-beam; THG, third harmonic generation of the pump-beam; SCG, supercontinuum white light generation for the probe-beam; DL, delay line; L, lens; P, polarizer; CR rotating compensator; A, analyzer. The repetition rate of the pump is 250 Hz.
